# Incorporation of a GPI-anchored engineered cytokine as a molecular adjuvant enhances the immunogenicity of HIV VLPs

**DOI:** 10.1038/srep11856

**Published:** 2015-07-07

**Authors:** Hao Feng, Han Zhang, Jiusheng Deng, Li Wang, Yuan He, Shelly Wang, Roheila Seyedtabaei, Qing Wang, Laiting Liu, Jacques Galipeau, Richard W. Compans, Bao-Zhong Wang

**Affiliations:** 1 Department of Microbiology and Immunology, Emory Vaccine Center, Emory University, Atlanta, GA 30322, USA; 2 Department of Hematology and Medical Oncology, Winship Cancer Institute, Emory University, Atlanta, GA 30322, USA; 3 Department of Bioengineering, Henan University of Technology, Zhengzhou 450052, China

## Abstract

HIV vaccines should elicit immune responses at both the mucosal portals of entry to block transmission and systemic compartments to clear disseminated viruses. Co-delivery of mucosal adjuvants has been shown to be essential to induce effective mucosal immunity by non-replicating vaccines. A novel cytokine, GIFT4, engineered by fusing GM-CSF and interleukin-4, was previously found to simulate B cell proliferation and effector function. Herein a membrane-anchored form of GIFT4 was constructed by fusing a glycolipid (GPI)-anchoring sequence and incorporated into Env-enriched HIV virus-like particles (VLPs) as a molecular adjuvant. Guinea pigs were immunized with the resulting HIV VLPs through an intramuscular priming-intranasal boosting immunization route. The GIFT4-containing VLPs induced higher levels of systemic antibody responses with significantly increased binding avidity and improved neutralizing breadth and potency to a panel of selected strains, as well as higher levels of IgG and IgA at several mucosal sites. Thus, the novel GPI-GIFT4-containging VLPs have the potential to be developed into a prophylactic HIV vaccine. Incorporation of GPI-anchored GIFT4 into VLPs as a molecular adjuvant represents a novel approach to increase their immunogenicity.

After three decades of effort to understand the pathogenesis and outcomes of human immunodeficiency virus (HIV) infection, we are still facing the daunting fact that 34 million people are living with HIV-1 with approximately 2 million new infections and 1.6 million deaths occurring every year^1^,^2^,^3^. Combination antiretroviral therapy (ART) has shown extraordinary success in reducing HIV transmission and prolonging life. However, ART treatment does not fully restore immune health, and a number of inflammation-associated and/or immunodeficiency complications can persist lifelong^4^. The vast majority of infected people in developing countries do not have access to antiviral drugs. A prophylactic vaccine remains the top priority for resolving the HIV-related challenges and problems.

Most recent endeavors for HIV vaccine development have been focused on eliciting broadly neutralizing antibody and T-cell responses. However, progress in HIV and SIV pathogenesis studies demonstrated that the early stage of infection at the mucosal portal of entry is a bottleneck for viral infection, and potential viral vulnerabilities were identified at this stage^5^,^6^. If founder populations of infected cells do not expand sufficiently to establish a self-propagating infection, the virus is at risk of elimination^7^. Considering the small founder populations revealed in SIV mucosal transmission studies and inferred in HIV transmission, this stage provides the maximal opportunity for vaccine-mediated disease prevention. Most HIV infections are acquired through sexual transmission^8^. Because HIV uses immune cells as hosts directly and integrates proviral DNA into the genomes of target cells, mucosal vaccines, rather than vaccines that are designed to limit virus load are more likely to completely prevent disseminated infection^8^.

A number of studies have suggested a promising role of cytokines as effective adjuvants to enhance immune responses^9^,^10^,^11^,^12^. In rhesus macaques, a heterologous prime-boost vaccine regimen with co-expression of GM-CSF and vaccine antigens conferred better protection against SIV infection compared to a non-adjuvanted group. This was correlated well with the elevated avidity of the elicited Env-specific IgG antibodies in the adjuvant treated group^13^,^14^. It was suggested that GM-CSF may enhance antibody avidity by recruiting and stimulating the maturation of antigen presenting cells, especially the myeloid-lineage dendritic cells^13^,^14^. GM-CSF and IL-4 together have been shown to induce the differentiation of monocytes into dendritic cells^15^,^16^,^17^. IL-4 has been shown to play a pivotal role in regulating precursor T-helper cell differentiation into the Th2 lineage and therefore facilitate humoral immune responses^18^.

Our previous studies have demonstrated that genetically modified Env proteins can be incorporated into virus-like particles (VLPs) with significantly improved efficiency^19^. The incorporated Env protein retains its conserved epitopes and probably the native conformation when incorporated into the VLPs^19^. Based on these findings, membrane-anchored flagellin was constructed and co-incorporated into VLPs as an adjuvant. Resulting HIV chimeric VLPs (cVLPs) elicited augmented neutralizing antibody and mucosal responses, further indicating the importance of a co-incorporated adjuvant into Env-enriched VLPs for eliciting both systemic and mucosal immune responses^3^. In a recent study, we found that a fusokine (fusion protein from two cytokines) from GM-CSF and IL4 (designated as GIFT4) leads to novel B-cell effector function manifest by an altered pro-immune cytokine secretory profile and a robust B-cell mitogenic response^20^. In the present study, we generated a membrane-bound form of GIFT4 by fusing the CD59 glycolipid (glycosylphosphatidyl-inositol, GPI) signal sequence to the GIFT4 C-terminal sequence in frame, and incorporated the membrane-anchored GIFT4 into Env-enriched VLPs. We found that these cVLPs harboring both a high density of Env and membrane-anchored GIFT4 elicited highly augmented Env-specific antibody responses with improved quality, as reflected by enhanced avidity and neutralization activity. These data demonstrate that the cVLPs have the potential to be further developed into an HIV mucosal vaccine.

## Results

### Co-incorporation of GPI-GIFT4 into cVLPs

**Figure 1:**
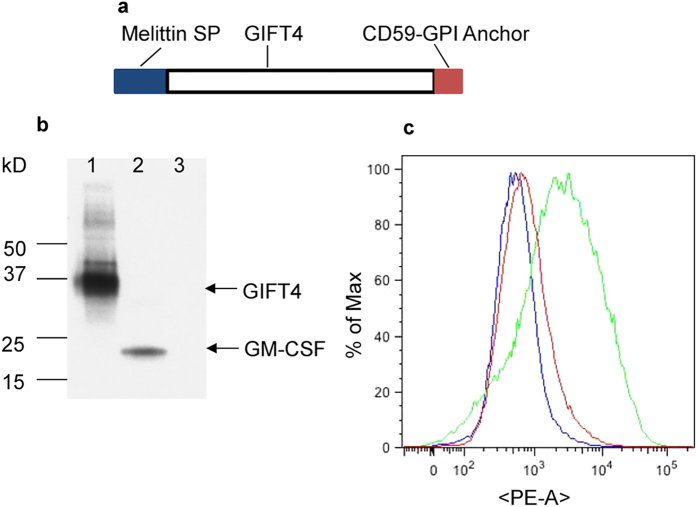
Diagram of GPI-anchored form of GIFT4 and insect cell expression. **a**, a schematic presentation of the GPI-GIFT4 encoding gene. Coding sequences for the melittin SP and murine CD59-GPI anchor were fused at the 5′ and 3′-ends of GIFT4 encoding DNA, respectively, to form the full-length gene. **b**, GPI-GIFT4 cellular expression. Western blots were developed with anti-GM-CSF antibody. Lane 1, whole cell lysate of GPI-GIFT4-expressing rBV infected insect cell sample; Lane 2, purified GM-CSF protein; Lane 3, whole cell lysate of HIV Env-expressing rBV infected cells as a control. **c**, FACS analysis of GPI-GIFT4 cell surface expression in rBV-infected insect cell samples. Red, isotype antibody control; Blue, Control Cells + anti-GMCSF antibodies; Green, GPI-GIFT4–expressing cells + anti-GMCSF antibodies.

A diagram of the membrane-bound form of GIFT4 gene is shown in Fig. 1a. The melittin signal peptide (SP) and CD59 GPI anchoring signal-coding sequences were fused to 5′ and 3′-ends of the GIFT4 encoding sequence in frame to facilitate the membrane insertion of GPI-GIFT4^21^. Western blot (Fig. 1b) using anti-GM-CSF antibody detected a band migrating at 37 kDa in the lysate of sf9 cells infected by recombinant baculoviruses (rBVs) expressing the GPI-GIFT4 gene (lane 1 in Fig. 1b), corresponding to the expected size of GPI-anchored GIFT4. The membrane anchoring of the expressed GPI-GIFT4 was further demonstrated by the enhanced fluorescent intensity measured by FACS analysis of the rBV-infected cells after cell surface staining with anti-GM-CSF antibodies, followed by PE-conjugated secondary antibodies (Fig.1c).

**Figure 2:**
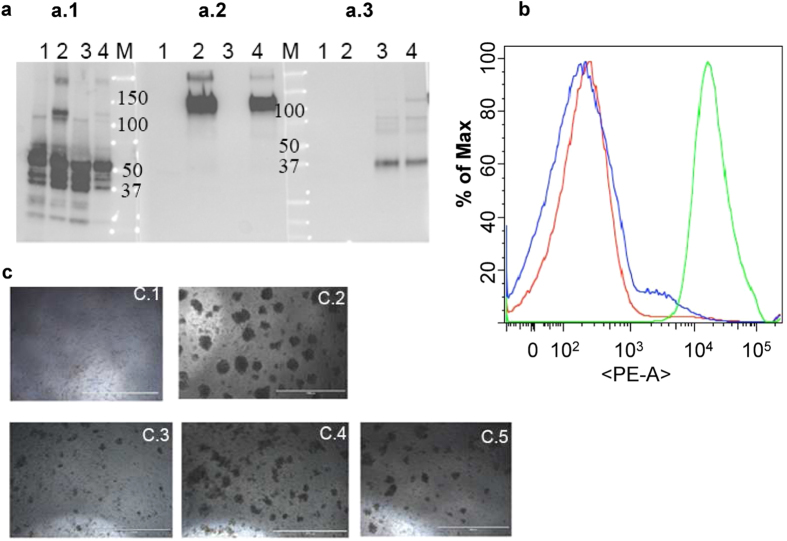
Characterization of VLPs. **a**, Western blotting analysis of the protein components of VLPs. VLP samples containing 1 μg of total protein were loaded for SDS-PAGE followed by western blotting. Protein bands were probed with: **a.1**, anti-HIV Gag antibody; **a.2**, anti-gp120 polyclonal antibodies; **a.3**, anti-GM-CSF antibody. Lane 1, Gag only VLPs; Lane 2, sVLPs; Lane 3, GIFT4/Gag VLPs; lane 4, cVLPs; M, Molecular weight (kDs). **b**, FACS analysis of GIFT4 GPI anchoring into VLPs using anti-GM-CSF antibody. VLP samples (1 ml at 1 mg/ml) were used for antibody staining as described for cell surface staining in Figure 1. Blue, sVLPs; Green, cVLPs; Red, cVLPs post-PIPLC treatment. **c**, proliferation of guinea pig splenocytes *in vitro* stimulated by VLPs or GIFT4. Spleen cells isolated from guinea pig were cultured *in vitro* for 2 days in the presence of 1 μg/ml of various HIV VLPs or the soluble GIFT4 (50 ng/ml) and photographed under an EVOS microscope. **C.1** to **c.5**, cells treated with PBS, soluble GIFT4, sVLPs, GIFT4/Gag VLPs, or cVLPs, respectively. Scale bar, 1000 μm.

VLPs were produced using the rBV expression system in insect cells. The protein composition of the resulting VLPs was characterized by western blot using antibodies specific to Gag, Env or GM-CSF. As shown in Fig. 2a (a.2), the Env incorporated into standard Env/Gag VLPs (sVLPs, lane 2) or cVLPs (lane 4) was observed to have a molecular mass of about 120 kDa^19^. A band migrating at the expected size of GPI-GIFT4 was seen in cVLP and GIFT4/Gag VLPs (lanes 3 and 4 in a.3), demonstrating the incorporation of GPI-GIFT4 into these VLPs. The results shown in lane 4 of both a.2 and a.3 in Fig. 2a further indicate that membrane-anchored GIFT4 and Env can be co-incorporated into HIV cVLPs. To verify that the integration of GIFT4 into VLPs is through GPI anchoring on the membrane surface, FACS assay was carried out. GIFT4 was detected by the enhanced fluorescent intensity in cVLPs (green curve in Fig. 2b) but not sVLPs (blue curve in Fig. 2b) after anti-GM-CSF antibody staining. Further, the GIFT4 signal from cVLPs was completely eliminated by treatment with PIPLC, a phosphatidylinositol-specific phospholipase which releases GPI-anchored molecules from membranes^22^, as shown in Fig. 2b (red curve). Together, these data demonstrated that GIFT4 can be incorporated into VLPs, or co-incorporated into cVLPs, through GPI anchoring.

### Biological function of GPI-GIFT4 in VLPs

To determine whether GPI-GIFT4 incorporated into VLPs still retained functional activity, we tested the potency of cVLPs in inducing proliferation of guinea pig spleen lymphocytes *in vitro*. As shown in Fig. 2c, after culturing for 2 days in the presence of 1 μg/ml of cVLPs or GIFT4/Gag VLPs, significantly higher numbers of spleen cells proliferated into colonies with larger colony sizes (c.4 and c.5) when compared to the control (c.1) or sVLPs (c.3)., Proliferation was also observed in sVLP-treated cells (c.3), although at a lower level when compared to that of the GIFT4 containing VLPs (c.4 and c.5 in Fig. 2c), demonstrating that VLPs themselves are also lymphocyte stimulators. These results indicate that GPI-GIFT4 incorporated into VLPs retains the biological activity of the soluble GIFT4 in stimulation of lymphocyte proliferation^20^.

### Enhanced systemic antibody responses to cVLPs

**Figure 3:**
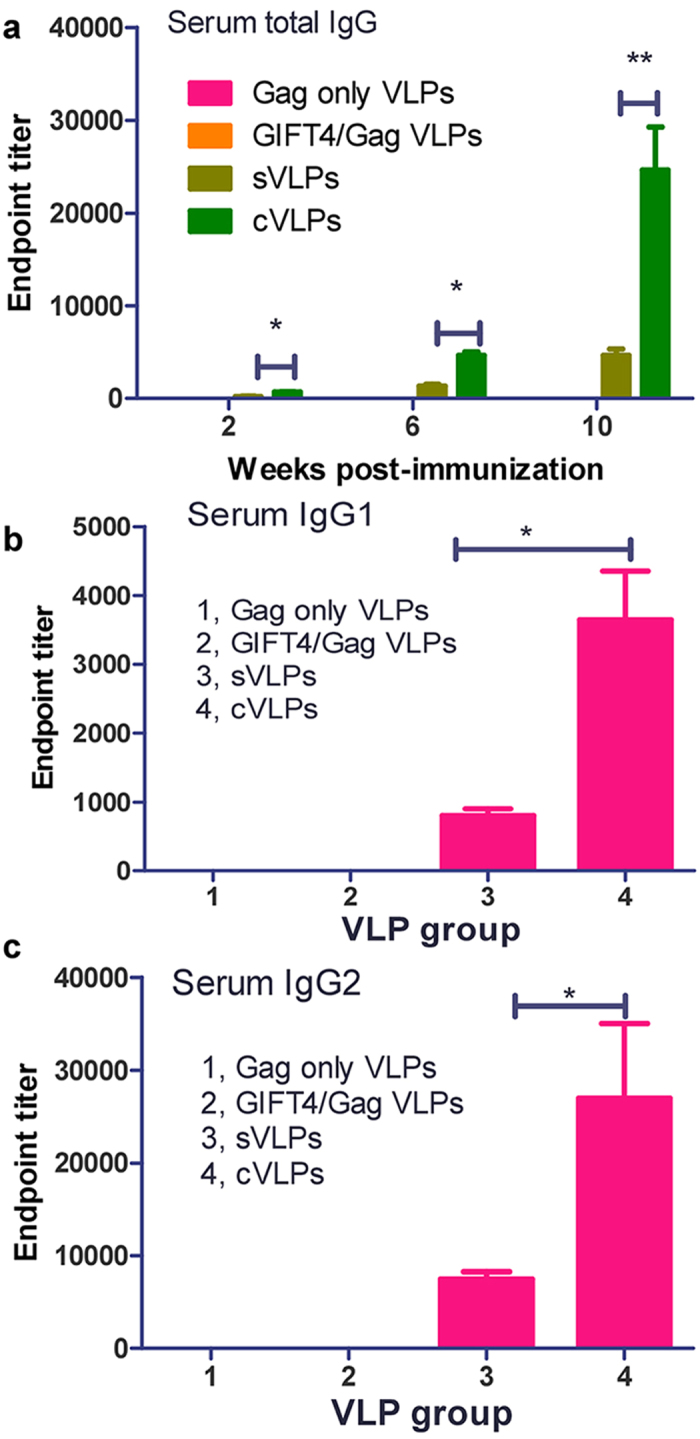
Serum IgG and IgG subtype endpoint titers. Guinea pigs were immunized with Gag only VLPs, GIFT4/Gag VLPs, sVLPs, or cVLPs in the one i.m. priming-two i.n. boosting route at weeks 0, 4, 8, respectively, and immune sere were collected 2 weeks after each immunization at weeks 2, 6, 10, respectively. **a,** Serum IgG endpoint titers; **b,** IgG1 titers of immune sera from bleed 3 (week 10); **c,** IgG2 endpoint titers of immune sera from bleed 3. Assays were performed as described in Materials and Methods. Results are expressed as means ± standard deviations. Student *t*-test was used for statistical analysis. The analysis was done by using GraphPad Prism version 5.00 for Windows (San Diego, California). P values of less than 0.05 (P < 0.05) were considered to be statistically significant. *P < 0.05; **P < 0.01.

To investigate whether GIFT4 incorporated into HIV VLPs enhances antibody responses against the Env immunogen, groups of guinea pigs were immunized with one intramuscular (i.m.) prime followed by two intranasal (i.n.) boosts with sVLPs, cVLPs, GIFT4/Gag VLPs or Gag only VLPs, respectively. Immune serum IgG levels specific to HIV Env at 2 weeks after each immunization were assessed by ELISA. The results shown in Fig. 3a (presented as endpoint titers) demonstrate that cVLPs induced serum antibody responses with higher titers than those observed with sVLPs (P < 0.05). After three immunizations (bleed 3 at week 10, Fig. 3a), guinea pigs immunized with cVLPs exhibited 5-fold higher IgG levels than those induced by sVLPs (means of 24600 *vs.* 4666, P < 0.01). These results indicated that co-incorporation of the membrane-anchored GIFT4 into VLPs is highly effective in enhancing anti-Env immune responses. Although cVLPs induced elevated IgG responses, Env-specific IgA in immune sera was not detected.

We assessed the serum IgG subclass profiles in the bleed 3 sera, and observed that sVLPs and cVLPs induced both IgG1 and IgG2 immune responses. The IgG2/IgG1 ratio for the sVLP group is about 9, and about 7 for the cVLP group (Fig. 3b and c). Based on these data, we concluded that IgG2 dominates the IgG responses to HIV VLPs. Compared to IgG1 and G2, G3 is much lower although IgG3 was not measured in this study due to the lack of a commercial anti-IgG3 antibody. Different to human IgG subtypes including G1, G2, G3 and G4, guinea pigs do not have subtype IgG4. Although cVLPs induced higher IgG titers compared to sVLPs, their antibody responses show similar IgG subtype profiles.

The sequence of GIFT4 used was derived from mice^20^. Thus we wanted to know whether antibody responses specific to GIFT4 were induced in guinea pigs, and whether these antibodies decrease the adjuvant function of GIFT4 in subsequent immunizations. However, we did not observe GIFT4-specific antibodies in immune sera (data not shown).

### Enhanced mucosal antibody responses to cVLPs

**Figure 4:**
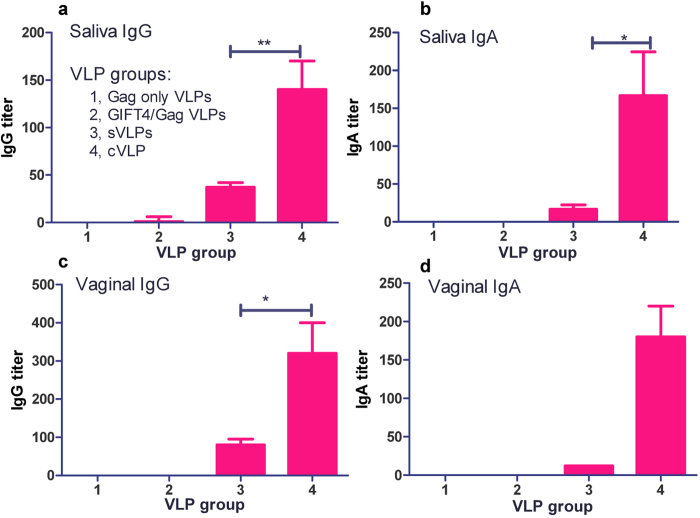
Mucosal antibody endpoint titers. Mucosal samples were collected at week 12, 4 weeks after the last boosting immunization. Env-specific IgG and IgA endpoint titers were detected by ELISA as described in Materials and Methods. **a**, saliva IgG; **b**, saliva IgA; **c**, v**a**ginal IgG; **d**, Vaginal IgA. Data were depicte**d** as means ± standard deviations. *P < 0.05; **P < 0.01.

It is well established that mucosal immunity is essential for controlling a primary HIV-1 infection. In the present study, we employed an immunization regimen that contained one i.m. prime supplemented by two i.n. boosts, which was expected to elicit enhanced mucosal immune responses. To determine whether cVLPs induce enhanced mucosal immune responses by this immunization regimen, we evaluated the secretory Env-specific IgA and IgG levels in saliva and vaginal washes after three immunizations. As shown in Fig. 4a,b, both Env-specific IgG and IgA titers in saliva samples were found to be much higher in the cVLP group than that in the sVLP group. Remarkably, at week 10, cVLP-immunized guinea pigs also showed about 5-fold higher IgG levels (Fig. 4c) and 6-fold higher IgA levels (Fig. 4d) in vaginal washes than those induced in sVLP-immunized guinea pigs, demonstrating that the GIFT4 is an effective adjuvant for eliciting mucosal immune responses.

### Enhanced antibody avidity

**Figure 5:**
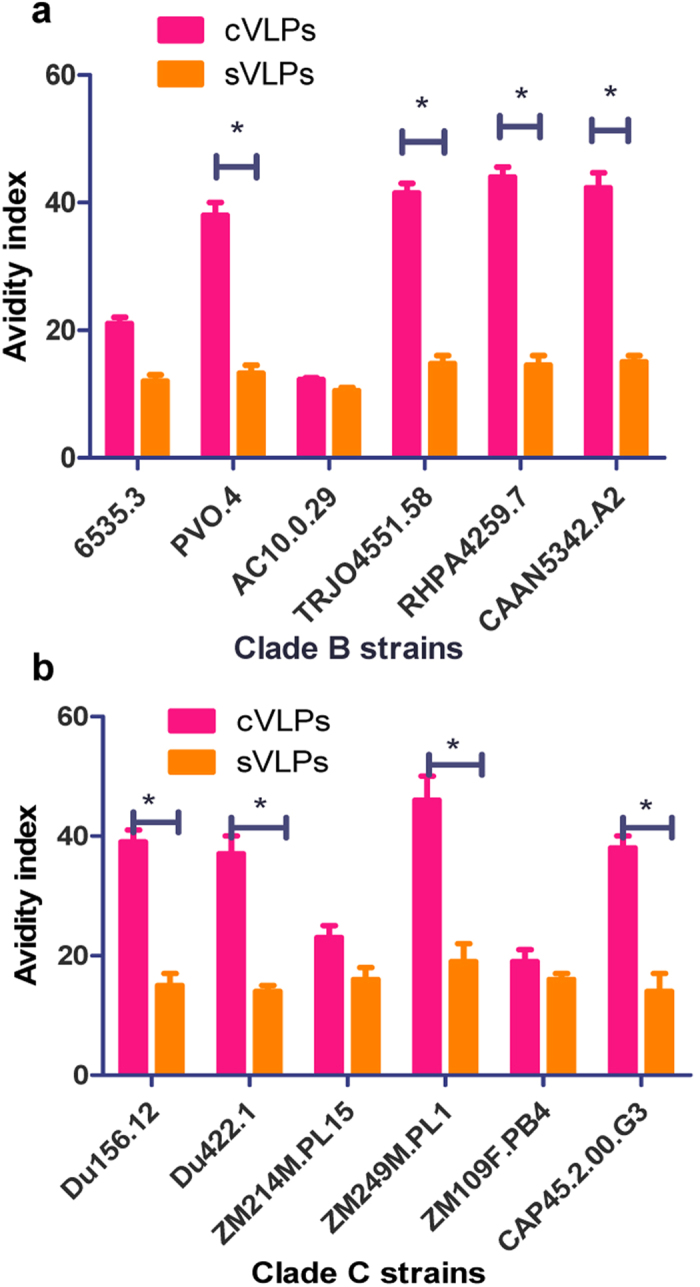
Avidity of immune serum IgG to selected pseudoviruses. Avidity assays were conducted with immune sera of bleed 3 (at week 10) from sVLP and cVLPs-immunized guinea pigs. **a**, avidity indexes of immune serum IgG to clade B pseudoviruses; **b**, avidity indexes of immune serum IgG to clade C pseudoviruses. Data are expressed as means ± standard deviations. *P < 0.05.

Antibody avidity for the HIV antigen is low at the early stage of infection and increases as the infection progresses while antibody matures^23^. Neutralizing antibodies with increased avidity evolve during maturation. A significant increase in avidity has been reported after repeated antigen exposure^24^. Several recent studies have also shown correlations between the avidity of non-neutralizing antibodies and HIV protective efficacy^25^,^26^,^27^. Therefore, antibody avidity analysis is an effective way to evaluate antibody quality for providing protection. To determine whether cVLPs induce antibody responses to Env with enhanced avidity, six Env-pseudotyped viruses from both clades B and C, were compared. Because Env is inserted into the pseudoviral envelope, as is the case in virions, Env in pseudoviruses and virions are functionally equivalent, binding to target cells and mediating virus-host cell membrane fusion. Pseudotyped virus-based neutralizing assays have been extensively used to evaluate an antibody capacity for blocking HIV infection. Thus antibody avidity to pseudovirus Env should reflect the antibody binding to HIV particles. The results shown in Fig. 5 demonstrated that serum antibodies in the cVLP group showed significantly increased avidity compared to the sera from the sVLP immunized group. As shown in Fig. 5, the cVLPs elicited antibodies with increased avidity with AIs around 40 for binding to 4 of the 6 clade B strains (Fig. 5a) as well as 4 of 6 clade C viruses (Fig. 5b), compared with sVLPs with AIs of no more than 20 (P < 0.05). Intermediate levels of avidity enhancement were found to strain 6535.3 in clade B and ZM214M.PL15 in clade C, and no change was observed with AC10.0.29 in clade B or ZM109F.PB4 (clade C) (P > 0.05). Interestingly, although cVLPs elicited increased avidity to sVLPs as observed above, avidity to Envs among these strains were not significantly different. Due to the much lower antibody levels in mucosal samples, avidity of both IgG and IgA in mucosal samples was not detectable, as is the case for serum IgA.

### Enhanced antibody neutralizing breadth and potency

**Figure 6:**
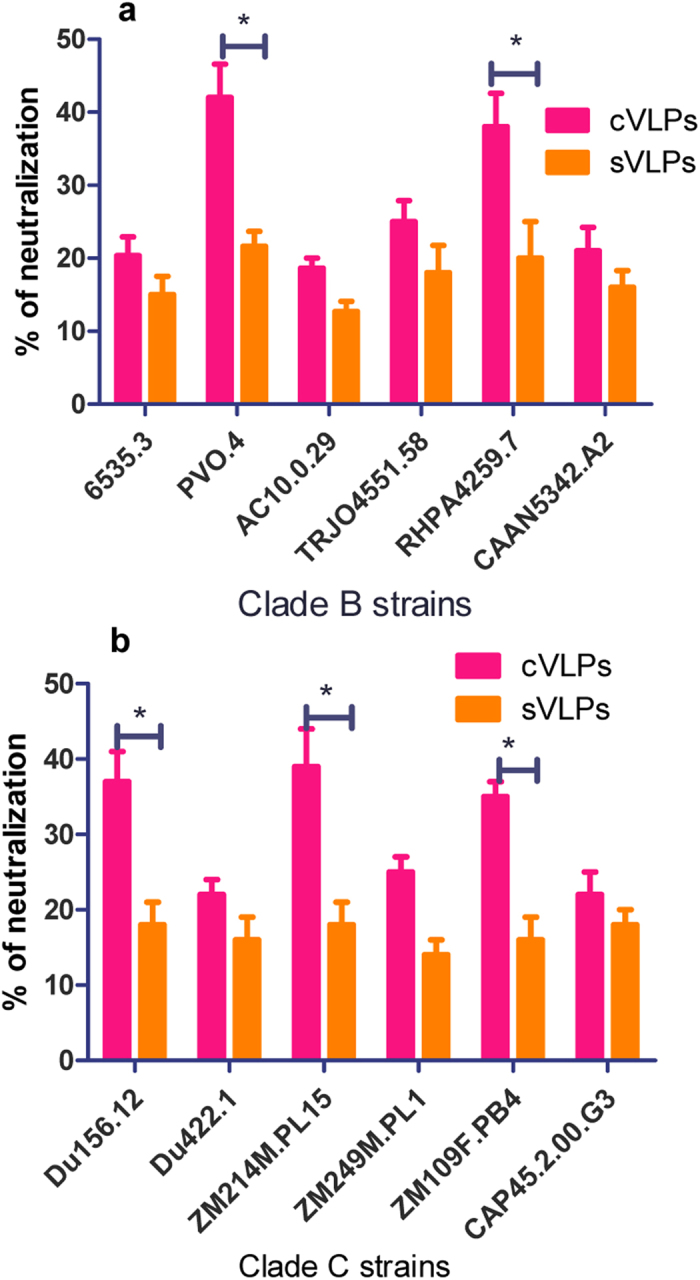
Neutralizing activity. Immune sera of the bleed 3 from sVLPs and cVLP-immunized animals were tested for neutralizing activity. The final dilution factor of immune sera was 40-fold. **a**, neutralization against clade B pseudoviruses. **b**, neutralization **a**gainst clade C pseudoviruses. Data were expressed as means ± standard deviations. *P < 0.05.

Neutralizing antibodies can directly block viral infection by binding tightly to the functional Env, mediating entry inhibition, virus aggregation, complement-dependent inactivation, or triggering antibody-dependent cell-mediated cytotoxicity/virus inhibition (ADCC/ADCVI)^28^,^29^,^30^, and thus are ideal targets to be elicited by a vaccine. Our results demonstrate that HIV cVLPs containing GPI-anchored GIFT4 induced higher titers of IgG compared to sVLPs. We further investigated the neutralization reactivity of these antibodies using a panel of HIV clade B and C Env-pseudoviruses, the same virus panel as was used to compare antibody binding avidity in Fig. 5. As shown in Fig. 6a, serum neutralizing reactivity elicited by the cVLP group against PVO.4, a tier 3 virus which shows strong resistance to neutralization^31^, and RHPA4259.7 (tier 2) were enhanced (approximately 30%–40% of the viruses were neutralized to lower than 20, P < 0.05) compared to the sVLP group. Of the 6 clade C viruses tested, immune sera from the cVLP group exhibited enhanced neutralization to Du156.12 (tier 2), ZM214M.PL15 (tier 2) and ZM109F.PB4 (intermediate) compared to the sVLP group (P < 0.05) (Fig. 6b). GIFT4-containing VLP and sVLP groups showed similar neutralization titers to the other viruses (P > 0.05). These results further indicate an adjuvant effect of the membrane-anchored GIFT4 in cVLPs in inducing antibody responses with enhanced neutralizing breadth and potency.

## Discussion

A major goal of vaccines against HIV is to elicit immune responses at the level of the mucosal surface to block transmission, as well as in systemic compartments to clear disseminated viruses. Vaccines administered by systemic routes generally fail to stimulate strong mucosal immune responses. In the present study, we developed cVLPs with efficient co-incorporation of HIV Env together with a novel fusokine, GIFT4, as a co-stimulatory factor, into the same particles. The resulting cVLPs were evaluated for their capacity to elicit enhanced antibody responses in systemic compartments as well as mucosal surfaces. This study integrates several approaches to enhance HIV immune responses. These include: 1) enhanced incorporation of Env into VLPs to increase the density of immunogens; 2) construction and co-incorporation of a membrane-bound form of GIFT4 into cVLPs to further improve immunogenicity by stimulating B lymphocyte proliferation and activation; 3) employment of a systemic prime/mucosal boost route to induce systemic as well as mucosal immune responses. Based on the present results, this integrated HIV vaccine strategy has the potential to be further developed to produce a prophylactic HIV vaccine in future.

Most current vaccines are administered by intramuscular injection, and induce systemic immune responses. However, mucosal immunity is rarely induced by systemic immunization. Since mucosal transmission is the predominant pathway for HIV infection and accounts for as high as 80% of AIDS incidence globally^32^, immunity functioning at the mucosal portals of entry is extremely important for preventing primary HIV-1 infection. Because of the relatively low efficiency of HIV infection at mucosal surfaces, even a modest enhancement of antibody-mediated protective mucosal immune responses could have a significant effect on reducing disease incidence^7^. Thus an effective HIV vaccine may need to induce both mucosal immunity to reduce the frequency of initial infection and possibly block the escape of virus from the genital and intestinal mucosa into systemic lymphoid organs, and systemic immunity, such as broadly neutralizing antibody responses, to clear any disseminated virus. In the present study, the intramuscular prime-intranasal boost immunization route was employed to induced enhance immunity both at systemic compartments and mucosal surfaces.

Previous studies have suggested the importance of the nasal cavity for induction of both mucosal and systemic immune responses due to the presence of germinal centers containing B, T, plasma, and professional antigen presenting cells (APCs)^33^. It is also well established that lymphoid tissues strategically positioned at the site of entry of the respiratory and the digestive tracts are important in antigen uptake. By two i.n. boosts with the newly constructed cVLPs, highly elevated mucosal HIV Env-specific IgG and IgA levels were induced compared to sVLPs administered by the same route. The antibodies secreted into the lumen are known to provide an immunological barrier to limit the penetration of antigens into mucous membranes and are strongly associated with protection from HIV-1 infection^34^,^35^,^36^. Although robust mucosal IgA and IgG responses were observed in animals immunized with cVLPs, serum IgA was not detectable. Because IgG is predominant over IgA in immune sera and may compete out IgA in binding to the same epitope in coating antigens in the ELISA, undetectable IgA may indicate that this antibody is either absent or in low titers in immune sera. In a recent analysis of immune correlates in the RV144 trial, serum IgA levels were found to be inversely correlated to HIV protection^37^. Low serum IgA levels in immune sera may therefore be an advantageous consequence of GIFT4-containing VLP-induced immunity.

An ideal antibody response should include not only high titers of antibodies but also high antibody avidity as well as neutralizing activity^24^,^25^,^26^,^27^. We observed enhanced antibody avidity and neutralizing breadth and potency in the cVLP-immunized group, supporting the conclusion that the adjuvant effect of GPI-GIFT4 enables the immune system to more effectively process or present B cell epitopes residing in Env. Although a correlation between antibody avidity and neutralization was not evident, the enhanced avidity observed may be contributed by non-neutralizing antibodies. Other recent studies have also shown correlations between the avidity of non-neutralizing antibodies and HIV protective efficacy^25^,^26^,^27^. The other different mechanisms may also be involved in the antiviral function of immunity such as ADCC and T cell response at mucosal sites including gut and rectal/vaginal mucosal tissues, to prevent the virus entry, contain the replication of escaped virus and delay the setting of a systemic infection^38^,^39^,^40^,^41^,^42^ . The study focused mainly on antibody responses (levels, avidity and neutralizing potency) because the gain-of-function activity of GIFT4 was found to facilitate B cell responses^20^.

GM-CSF has been proven to be an effective adjuvant for HIV vaccines in a number of studies. In rhesus macaques, anti-Env antibodies with improved avidity were elicited by co-delivery of GM-CSF DNA with Gag, Pol, and Env DNA followed by an MVA boost^13^. The adjuvanted group exhibited better control of challenge and re-emergent virus infection^13^. In addition, APCs are important to induce a robust immune response. Previous studies have shown that GM-CSF combined with IL-4 optimizes the growth of human DCs *in vitro*^17^. Both cytokines are known to promote differentiation and maturation of non-growing human blood monocytes or proliferating bone marrow-derived precursors^43^. The mechanisms underlying the enhanced immune responses seen in the aforementioned as well as the present studies may be closely related to the role of GM-CSF and IL-4, or its gain-of-function of GIFT4, in promoting differentiation and maturation of monocytes into dendritic cells (DC)^16^. Different from single cytokines or their mixture, engineered fusokines can generate novel functions as stimulators of lymphocytes as previously demonstrated^20^,^44^,^45^. GIFT4 was found to generate gain-of-function activity to induce robust B cell proliferation and lead to enhanced B cell responses^20^. By further coupling a GPI anchor to allow GIFT4 to be co-incorporated into VLPs together with Env, the integrated antigen-stimulator in the same cVLPs may provide targeted immune cells for better antigen presentation and proliferation. The enhanced adjuvant efficacy by co-incorporation of an immune stimulator into VLPs, compared with mixing with VLPs, has also been demonstrated previously^46^,^47^,^48^. Robust proliferation of Env-primed B cell populations stimulated by GIFT4 may be an important factor for the enhanced antibody responses in cVLPs-immunized animals in the present study. Furthermore, because GIFT4 is derived from cytokines (GM-CSF and IL-4), it is highly safe when used as a molecular adjuvant.

In this study, the great value of GPI-anchored GIFT4 in VLPs as a novel adjuvant is supported by improved systemic as well as mucosal antibody responses with augmented avidity and broadened neutralization capacity. These data demonstrate the potential of GIFT4-containing HIV VLPs for development of an effective HIV vaccine for administration by the i.m. prime-i.n. boosting immunization route. Incorporation of the membrane-anchored GIFT4 can be a general approach to improve the immunogenicity of various VLP vaccines.

## Methods

### Ethics Statement

This study was carried out in strict accordance with the recommendations in the Guide for the Care and Use of Laboratory Animals of the National Institutes of Health. All guinea pig studies were approved by the Emory University Institutional Animal Care and Use Committee (IACUC). The IACUC issued a protocol number of 079-2008Y for this study. Female guinea pigs (8-week old) were purchased from the Jackson Laboratory and housed in the animal facility at Emory University. Immunization and bleeding were performed under anesthesia that was induced and maintained with ketamine hydrochloride and xylazine, and all efforts were made to minimize suffering.

### Construction and expression of GPI-anchored GIFT4

To generate a gene encoding the membrane-anchored GIFT4, the coding sequences of the signal peptide from the honeybee melittin and murine CD59 GPI anchor were fused to the 5′- and 3′-ends of the GIFT4 coding gene (derived from mouse sequences^20^) in frame to obtain the full-length encoding gene of a GPI-anchored GIFT4 (GPI-GIFT4) by overlapping PCR^20^,^49^,^50^,^51^. The resulting GPI-GIFT4 encoding gene was then cloned into transfer vector pFastBac-1 plasmid (Invitrogen, Carlsbad, CA). A recombinant baculovirus (rBV) expressing GPI-GIFT4 was generated by using the Bac-to-Bac insect cell protein expression system (Invitrogen, Carlsbad, CA).

To confirm whether GPI-anchored GIFT4 can be membrane-oriented translocated and expressed on cell surfaces, sf9 cells were infected with rBVs expressing GPI-GIFT4 at a MOI of 2. Two days later, cells were harvested and stained with rat anti-mouse GM-CSF antibodies (BD Biosciences) followed by PE-conjugated secondary antibodies. A non-GIFT4-related rat anti-mouse antibody served as an antibody control. Sf9 cells infected with rBVs expressing Env were stained with anti-GM-CSF followed by PE-conjugated secondary antibodies as another control. Fluorescent intensity was recorded and analyzed by FACS with a BD FACSCanto II flow cytometer.

### Production of HIV VLPs

Four different VLPs (Gag only VLPs, GIFT4/Gag VLPs, sVLPs and cVLPs) were produced for comparison using an insect cell expression system as described previously^19^. For the production of cVLPs, sf9 cells were co-infected with three rBVs respectively expressing a modified HIV Env consensus (ConS) which showed a high level of incorporation into VLPs, GPI-GIFT4, and Gag, at MOIs of 6, 2 and 3, respectively^3^,^19^. Standard VLPs and Gag only VLPs were produced as described^19^. GIFT4/Gag VLPs were produced by co-infection of sf9 cells with rBVs expressing GPI-GIFT4 and Gag at MOIs of 2 and 3, respectively. Two days post-infection, the culture supernatant was collected and VLPs were concentrated by porous fiber filtration using the Quixstand benchtop system (GE Healthcare, Uppsala, Sweden) followed by sucrose density gradient ultracentrifugation as described previously^50^. To quantitate the yield of purified VLPs, the protein concentration of each sample was estimated using the Bio-Rad protein assay (Bio-Rad Laboratories, Inc, Hercules, CA).

### Functional characterization of GIFT4-contraining VLPs

To determine whether the anchored GIFT4 in cVLPs retains the biological activity of soluble GIFT4, we tested whether cVLPs can induce proliferation of guinea pigs spleen cells *in vitro*. The spleen cells were cultured in complete RPMI medium in the presence of 1 μg/ml sVLPs, cVLPs, or GIFT4/Gag VLPs, respectively. Soluble GIFT4 (50 ng/ml) was used as a positive control. Following incubation at 37 °C in 5% CO_2_ for 2 days, the proliferation of cells was observed and imaged under an EVOS microscope (Life Technologies, Grand Island, NY).

### Immunization of guinea pigs and sampling

Female Hartley strain guinea pigs were obtained from Charles River Laboratory (Wilmington, MA) and were separated into four groups (5 animals per group). Groups were immunized with an immunization regimen including one intramuscular (i.m.) prime followed by two intranasal (i.n.) boosts with VLP vaccines at intervals of 4 weeks. For each immunization, animals in the Gag only and GIFT4/Gag VLP groups were immunized with 100 μg total protein, respectively. Standard and cVLPs were administered using doses containing 10 μg Env, respectively. As averages, one dose of GIFT4-containing VLPs (cVLPs and GIFT4/Gag VLPs) contained about 2 μg GIFT4 calibrated by using soluble GIFT4. Two weeks after each immunization, immune sera were collected by vena cava bleeding of anesthetized guinea pigs.

### Mucosal sample collection

Mucosal samples were collected at week 12, 4 weeks after the last boosting immunization. Vaginal lavages were collected by lavaging 250 μl of PBS intravaginally with an oral feeding needle (Braintree Scientific Inc., Braintree, MA)^52^. To collect saliva samples, pre-weighed medical cotton Q-tips were inserted into the mouth and left for 5 min and then weighed and extracted with PBS plus 0.02% Tween 20. Samples were briefly centrifuged and the supernatants were filtered through a 0.22 μm filter and stored at −80 °C for further analysis.

### Evaluation of systemic and mucosal antibody responses

To determine HIV Env specific IgG, IgG1, IgG2 and IgA antibody responses, ELISA plates (Nunc-Immuno Plate MaxiSorp™, Nunc Life Technologies, Basel, Switzerland) were coated with ConS Env gp120 purified from 293T cells infected with a recombinant vaccinia virus^3^,^19^. The highest dilution factor that gives an OD 450 of twice that of the naïve sample at the dilution was designated as the antibody end point titer.

### Pseudovirus preparation and neutralization assays

Each of six *env* expression plasmids from an NIH standard reference panel for clade B and C, respectively, were selected to generated Env-pseudotyped viruses for binding avidity and neutralization assays. These tier 2/3 viruses included 6535.3, PVO.4, AC10.0.29, TRJO4551.58, RHPA4259.7 and CAAN5342.A2 in clade B and Du156.12, Du422.1, ZM214M.PL15, ZM249M.PL1, ZM109F.PB4 and CAP45.2.00.G3 in clade C^53^,^54^.

Pseudoviruses were prepared by transfecting 293T cells (6–8 × 10^6^ cells in 15 ml growth medium in a T-75 cell culture flask) with 10 μg of a specific *env* expression plasmid and 10 μg of the *env*-deficient HIV-1 backbone vector (pSG3△Env) using Lipofectamine 2000 (invitrogen). Pseudoviruses contained in the culture supernatants were harvested 2 days post transfection and filtered through a 0.45 μm filter. The 50% tissue culture infectious dose (TCID_50_) of each pseudovirus was measured and samples were stored at −80 °C for further analysis.

Neutralization assays were performed in JC53-BL cells as previously described with minor modification^3^. Briefly, JC53-BL cells were seeded at 25,000 cells per well to a 96-well plate 24 h prior to infection. Guinea pig serum samples were heat-inactivated at 56 °C for 30 min and diluted 1:20 in 10% FCS–DMEM to a final volume of 25 μl and added to 25 μl of pseudovirus stock diluted in 10% FCS–DMEM containing 50 infectious particles (final serum dilution, 1:40). Viruses mixed with medium only were used as controls. The virus-immune serum mixtures were incubated at 37 °C for 1 h and then added to JC-53 cells with DEAE-dextran at a final concentration of 15 μl /ml. After 2 h of incubation, an additional 200 μl of complete DMEM was added. Two days post-infection, the medium was removed and the cells were fixed and stained as described by Chackerian *et al*^55^. Antibody neutralizing activity is presented as percentage of neutralization and calculated as [(average number of blue foci in control wells-average number of blue foci in virus-serum mixture sample wells)/(average number of blue foci in control wells)] ×100%.

### Antibody avidity assay

96-well microtiter plates were coated with 100 μl of Env-pseudovirions (4 μg/ml) in coating buffer (0.1 M sodium carbonate, pH 9.5) at 4 °C overnight, the diluted serum samples were added to wells and incubated for 1.5 hours at 37 °C. The assay was conducted using parallel titrations of immune sera with or without treatment with 1.5 M sodium thiocyanate (NaSCN) for 15 min to discriminate weak binding from high-affinity binding between antibodies and antigens, following the binding of immune sera. The antibody titers were subsequently determined using the standard ELISA procedure. The avidity index (AI) was calculated by dividing the titer with NaSCN treatment by the titer without the NaSCN treatment and multiplying by 100^56^,^57^.

## Additional Information

**How to cite this article**: Feng, H. *et al.* Incorporation of a GPI-anchored engineered cytokine as a molecular adjuvant enhances the immunogenicity of HIV VLPs. *Sci. Rep.* **5**, 11856; doi: 10.1038/srep11856 (2015).
